# Functional Restoration of *BRCA1* Nonsense Mutations by Aminoglycoside-Induced Readthrough

**DOI:** 10.3389/fphar.2022.935995

**Published:** 2022-06-28

**Authors:** Renata B. V. Abreu, Thiago T. Gomes, Thales C. Nepomuceno, Xueli Li, Mateus Fuchshuber-Moraes, Giuliana De Gregoriis, Guilherme Suarez-Kurtz, Alvaro N. A. Monteiro, Marcelo A. Carvalho

**Affiliations:** ^1^ Divisão de Pesquisa Clínica, Instituto Nacional de Câncer, Rio de Janeiro, Brazil; ^2^ Cancer Epidemiology Program, H. Lee Moffitt Cancer Center and Research Institute, Tampa, FL, United States; ^3^ Instituto Federal do Rio de Janeiro—IFRJ, Rio de Janeiro, Brazil

**Keywords:** *BRCA1*, aminoglycoside, premature stop codon, PTC, nonsense mutation, readthrough

## Abstract

BRCA1 is a major tumor suppressor that functions in the accurate repair of DNA double-strand breaks via homologous recombination (HR). Nonsense mutations in *BRCA1* lead to inactive truncated protein products and are associated with high risk of breast and ovarian cancer. These mutations generate premature termination codons (PTCs). Different studies have shown that aminoglycosides can induce PTC suppression by promoting stop codon readthrough and restoring full-length (FL) protein expression. The use of these compounds has been studied in clinical trials for genetic diseases such as cystic fibrosis and Duchenne muscular dystrophy, with encouraging results. Here we show proof-of-concept data demonstrating that the aminoglycoside G418 can induce *BRCA1* PTC readthrough and restore FL protein synthesis and function. We first demonstrate that G418 treatment restores *BRCA1* FL protein synthesis in HCC1395, a human breast tumor cell line carrying the R1751X mutation. HCC1395 cells treated with G418 also recover HR DNA repair and restore cell cycle checkpoint activation. A set of naturally occurring *BRCA1* nonsense variants encoding different PTCs was evaluated in a GFP C-terminal BRCA1 construct model and *BRCA1* PTC readthrough levels vary depending on the stop codon context. Because PTC readthrough could generate FL protein carrying pathogenic missense mutations, variants representing the most probable acquired amino acid substitutions in consequence of readthrough were functionally assessed by a validated transcription activation assay. Overall, this is the first study that evaluates the readthrough of PTC variants with clinical relevance in the breast and ovarian cancer-predisposing gene *BRCA1*.

## Introduction

Germline mutations in the tumor suppressor gene *BRCA1* (Breast Cancer 1, early onset) are associated with an increased lifetime risk for familial breast and ovarian cancers (King et al., 2003). Pathogenic variants may confer an average cumulative risk of up to 72% for breast and 44% for ovarian cancers by the age of 80 years (Kuchenbaecker et al., 2017). The *BRCA1* gene encodes the homonymous protein that includes two characteristic domains, a RING-finger at the N-terminus and the tandem BRCT (tBRCT) at the C-terminal end (Koonin et al., 1996; Tarsounas and Sung, 2020). The tBRCT is known to present sequence-specific binding to phosphorylated peptides critical to BRCA1 tumor suppression function (Shakya et al., 2011). BRCA1 is responsible for maintaining genomic integrity, being a key player in DNA double-strand breaks (DSBs) repair via homologous recombination (HR) and cell cycle checkpoint signaling (Prakash et al., 2015; Billing et al., 2018). Mutations that disrupt the tBRCT structure are functionally classified as pathogenic and the loss of the last 7 amino acids residues of the C-terminus abrogates the tBRCT function and increases cancer risk (Fernandes et al., 2019).

To date (2022), more than 34,000 unique *BRCA1* variants are reported in the BRCA Exchange platform (https://brcaexchange.org/factsheet, Cline et al., 2018)) and they are distributed throughout the gene sequence (Golubeva et al., 2019). In the clinical database ClinVar (https://www.ncbi.nlm.nih.gov/clinvar), *BRCA1* nonsense mutations account for ∼20% of variants classified as pathogenic. Nonsense mutations generate premature termination codons (PTC) resulting in truncated and dysfunctional protein products.

PTC suppression (readthrough) has been studied as a potential therapeutic strategy for genetic diseases caused by nonsense mutations, notably cystic fibrosis (CF) and Duchenne muscular dystrophy (DMD) (Martins-Dias and Romão, 2021). PTC readthrough therapies imply the incorporation of a near-cognate amino acid in the polypeptide chain instead of interrupting the synthesis at the premature stop codon. As consequence, the wild-type protein may be restored but also a missense variant can be generated leading to a product of unknown functional significance (Roy et al., 2016; Wangen and Green, 2020).

The first pharmacological approach to induce PTC suppression in mammalian cells used aminoglycosides, such as geneticin (G418) and gentamycin (Burke and Mogg, 1985; Howard et al., 1996). Since then, aminoglycosides have been widely studied as PTC suppressors in genetic diseases (Lee and Dougherty, 2012; Martins-Dias and Romão, 2021), but *in vivo* toxicity of these compounds limits treatment setups (Guthrie, 2008; Wargo and Edwards, 2014). Other small molecules have also been studied as PTC readthrough inducers, highlighting PTC-124 (Ataluren®), already tested in human clinical trials for CF and DMD patients carrying nonsense mutations (Wilschanski et al., 2011; Kerem et al., 2014; McDonald et al., 2017). Recently, translation termination factors inhibitors have been identified as prominent agents to potentiate the effect of readthrough inducing compounds (Baradaran-Heravi et al., 2021; Sharma et al., 2021).

Aminoglycosides compose a class of antibiotics that targets the decoding site of the ribosome, promoting error-prone protein translation which can lead to complete inhibition of protein synthesis in bacteria (Davis, 1987). Due to the lower affinity for the eukaryotic ribosome, aminoglycosides induce PTC readthrough and restore full-length (FL) protein synthesis (Recht et al., 1999). The aminoglycoside-mediated readthrough usually leads to low levels of the FL protein, nevertheless, it is proven to recover functional protein levels in several nonsense-mediated genetic disorders such as CF, hemophilia A and β-Thalassemia (Rowe et al., 2011; Borgatti et al., 2020; Martorell et al., 2020).

Here, we sought to evaluate the effect of aminoglycoside G418 on *BRCA1* naturally occurring PTC mutations. First, we interrogate the effect of G418 treatment on a breast cancer cell line harboring a nonsense mutation in homozygosis. Furthermore, we investigate the influence of PTC sequence context in *BRCA1* readthrough and the impact of predicted incorporated amino acids in BRCA1 function. Taken together, our results demonstrate proof-of-concept that PTC readthrough is a possible alternative to circumvent specific nonsense mutations in *BRCA1*.

## Materials and Methods

### Cell Culture and G418 Treatment

HCC1395 (ATCC CRL-2324™), Phoenix (ATCC CRL-3213™) and HeLa (ATCC CCL-2™) cells were cultured in RPMI 1640 medium (Gibco). HEK293FT (ATCC CRL-11268™) cell line was maintained in DMEM medium (Gibco). All media were supplemented with 10% v/v fetal bovine serum, 100 U/ml penicillin, and 100 μg/ml streptomycin (Sigma-Aldrich). Cells were cultured in a 5% v/v CO_2_ atmosphere at 37^o^C.

PTC readthrough was induced 24 h after cell seeding, with 300 μg/ml of G418 (Sigma-Aldrich) treatment for 48 h.

### Mutagenesis and Plasmid Constructions

*BRCA1* nonsense variants were generated using as template the pcDNA3:GAL4-BRCA1 WT 13/24 plasmid containing part of human wild-type (WT) *BRCA1* cDNA (GenBank accession U14680.1) that encodes amino acid residues 1,396 to 1,863 (corresponding to exons 13 to 24; *BRCA1* 13/24) with an in-frame fusion of GAL4 DNA binding domain (DBD) in the N-terminus (Fernandes et al., 2019). Variants were generated by site-directed mutagenesis using two different approaches: 1) whole plasmid amplification adapted from the GeneTailor^TM^ Site-Directed Mutagenesis System (Invitrogen) method. Briefly, the template plasmid was amplified by PCR using PrimeStar DNA polymerase (Takara Bio Inc.) and mutagenesis primers designed according to the kit instructions. PCR product was then treated with *Dpn*I and used to transform *E. coli* DH5α bacteria; 2) overlap extension as previously described (Carvalho et al., 2014). Primers used for mutagenesis are listed in Supplementary Table S1.

To generate the retroviral reporter vector pQCXIH:EGFP, EGFP coding sequence was amplified from pEGFP-C3 vector (Takara Bio Inc.) using specific primers (FW: 5′-CGA​CCG​GTA​TGG​TGA​GCA​AGG​GCG​AG and RV: 5′-CGT​TAA​TTA​ATT​ACT​ATC​AGT​TAT​CTA​GAT​C) and cloned into pQCXIH vector using *Age*I and *Pac*I restriction sites.

*BRCA1* WT 13/24 and nonsense variants coding sequences in pcDNA3:GAL4-BRCA1 13/24 were amplified by PCR using specific primers that do not include the natural stop codon of *BRCA1* gene (FW: 5′-CGG​CGG​CCG​CAT​GCA​GAG​GGA​TAC​CA and RV: 5′-CGA​CCG​GTG​TAG​TGG​CTG​TGG​GGG​AT). Then, amplicons were cloned in-frame with EGFP within the previous generated pQCXIH:EGFP construct using *Not*I and *Age*I restriction enzymes, generating pQCXIH:BRCA1-EGFP constructs.

All constructs were confirmed by Sanger direct sequencing. Restriction enzymes were purchased from New England Biolabs.

### Retroviral Transduction

Phoenix cells (6 × 10^6^) were transiently transfected with 20 µg of pQCXIH:BRCA1-EGFP constructs using PEI (poliethylenimine; Sigma-Aldrich) as previously described (Longo et al., 2013). Retroviral particles were collected from the supernatant 48 h after transfection. HeLa cells (3.5 × 10^3^) were transduced with the total fresh retroviral particles in the presence of 6 μg/μL polybrene (Sigma-Aldrich), followed by hygromycin B (200 mg/ml, Sigma-Aldrich) selection 24 h post-transduction. Stable cell lines (HeLa BRCA1-EGFP) were maintained under hygromycin B pressure for up to 72 h before readthrough assays.

### EGFP Flow Cytometry and Fluorescence Microscopy

HeLa BRCA1-EGFP cells (G418-treated or control) were harvested and resuspended in PBS for analysis. Flow cytometry was performed using BD Accuri C6 Flow Cytometer (BD Biosciences). Data analysis was performed using CFlow Plus (BD Biosciences).

For fluorescence microscopy, HeLa BRCA1-EGFP cells (5 × 10^4^) previously plated on glass coverslips and maintained in culture for 24 h were treated (or not) with G418. Cells were then washed with PBS and fixed in 4% w/v paraformaldehyde (Merck) prepared in PBS. Slides were mounted with ProLong^TM^ Gold Antifade Mountant with DAPI (Thermo Fisher Scientific). Cell images were captured and analyzed using FV10i-O confocal microscope and FV10-SW software (both Olympus).

### Transcription Activation Assay

BRCA1 fusion products were functionally assessed by TA assay, as previously described (Fernandes et al., 2019). Briefly, HEK293FT cells were seeded (3.5 × 10^4^) in a 96-well plate and after 24 h co-transfected (using PEI) with *BRCA1* constructs containing missense variants or controls (pcDNA3:GAL4-BRCA1 13/24), the pG5Luc plasmid enclosing *Photinus pyralis* luciferase reporter gene driven by GAL4 binding sites, and the phGR-TK plasmid (both Promega) which codes for the constitutively expressed *Renilla reniformis* luciferase (internal control). Cells were harvested 24 h post-transfection and the TA was measured using the Dual-Luciferase Reporter Assay System and the GloMax^TM^ 20/20 Luminometer (both Promega).

### Homologous Recombination Repair Assay

HR efficiency was assessed as described previously by (Nepomuceno et al., 2017), adapted from (Mao et al., 2009). In brief, HCC1395 cells were co-transfected (using PEI) with pDsRED2-N1 and the I-*Sce*I digested HR reporter plasmid (GFP-based). Twenty-four hours later, cells were treated with G418. Cells were harvested and resuspended in PBS for flow cytometry analysis using BD FACS Calibur (BD Biosciences). Data analysis was performed using FlowJo software (BD Biosciences) and GFP-positive cells were calculated relative to DsRed internal reporter control positive cells.

### Cell Cycle Analysis

HCC1395 cells (G418-treated or control) were exposed to ionizing radiation (IR; 6 Gy) and allowed to recover for 1 h. Cells were collected and fixed in 70% v/v ice-cold ethanol for 16 h, followed by 5% w/v BSA (in PBS) blocking for 30 min. Immunostaining was performed using mouse monoclonal α-phosphoH3_ser10_ (cat.no. 05–806; Merck) for 30 min, followed by Alexa488-conjugated α-mouse (cat.no. A-11029; Invitrogen) for 1 h. Cells were incubated with 1 mg/ml propidium iodide and 100 ug/ml RNAse A for 1 h (both Sigma-Aldrich). Incubations were conducted at room temperature and flow cytometry was conducted using BD FACSCanto^TM^ (BD Biosciences). Data analysis was performed using FlowJo software (BD Biosciences).

### Immunoblotting and Antibodies

Nuclear extracts were obtained as previously described (Rios-Doria et al., 2009). Briefly, cells were incubated in ice-cold buffer A (20 mm Tris pH 7.4, 10 mm KCl, 1 mm EDTA, 0.2% NP40, 50% glycerol, 600 mm β-mercaptoethanol) supplemented with protease inhibitors cocktail. Nuclei were recovered by centrifugation and incubated in ice-cold buffer B (20 mm Tris pH7.4, 10 mm KCl, 400 mm NaCl, 1 mm EDTA, 50% glycerol, 600 mm β-mercaptoethanol) supplemented with protease inhibitors cocktail for 30 min. Nuclear debris were removed by centrifugation. Whole cellular extracts were obtained by lysing cells in ice-cold mild-RIPA buffer (NaCl 150 mm, Tris-Cl 100 mm pH 7.4, EDTA 5 mm, NP40 1% v/v) supplemented with protease inhibitors cocktail for 30 min. Cellular debris were removed by centrifugation and supernatant was recovered. All chemicals were purchased from Sigma-Aldrich.

Immunoblotting was performed using PVDF membranes (Millipore) and developed using the ECL Plus kit (Amersham Biosciences). Mouse monoclonal α-GAL4 DBD (cat.no. SC-510T, Santa Cruz Biotech), rabbit polyclonal α-BRCA1 D-20 (cat.no. SC-641, Santa Cruz Biotech), rabbit polyclonal α-BARD1 (cat.no. SC-11438, Santa Cruz Biotech), HRP conjugated α-mouse IgG (cat.no. SC-2005, Santa Cruz Biotech) and rabbit monoclonal α-PCNA (cat.no. 13110, Cell Signaling technology) were used for immunoblotting. Mouse monoclonal α-BRCA1 Ab-1 (cat.no. OP92, EDM Millipore) and Rabbit polyclonal α-ABRAXAS (cat.no. A302-180A, Bethyl Laboratories) were used for co-immunoprecipitation assays.

### Co-Immunoprecipitation

Co-immunoprecipitation assays were performed by incubating 500 mg of nuclear extracts and the appropriate antibody for 16 h at 4°C in mild-RIPA. Immunocomplexes were bound to Protein A/G Plus Agarose beads (Santa Cruz Biotechnology) for 1 h at 4°C, followed by extensively ice-cold mild-RIPA buffer washes. The protein complexes obtained were analyzed by immunoblotting.

### Statistical Analysis and Align-GVGD

Data are expressed as mean ± standard deviation (SD). Statistical analysis was performed using GraphPad Prism 8.0 software (GraphPad Software Inc., California, United States). Data distribution was assessed by the Shapiro-Wilk normality test. F-test was used to compare the equivalence of two variances. One and two-sample Student’s t-test were used for the comparison of HR efficiency and cell cycle checkpoint on HCC1395 cells, respectively. The threshold for significance was set as *p* < 0.05.

Selected *BRCA1* missense variants were assessed by the web-based software Align-GVGD (https://agvgd.hci.utah.edu, (Mathe et al., 2006)) to predict their functional impact on protein biology. The program aligns ortholog sequences of the *BRCA1* protein (depth chosen: human to sea urchin) and determines the impact of a single amino acid substitution, scoring from C0 (less likely to interfere with function) to C65 (more likely to interfere with function).

## Results

### G418 Induces BRCA1 Restoration in HCC1395 Cell Line

**FIGURE 1:**
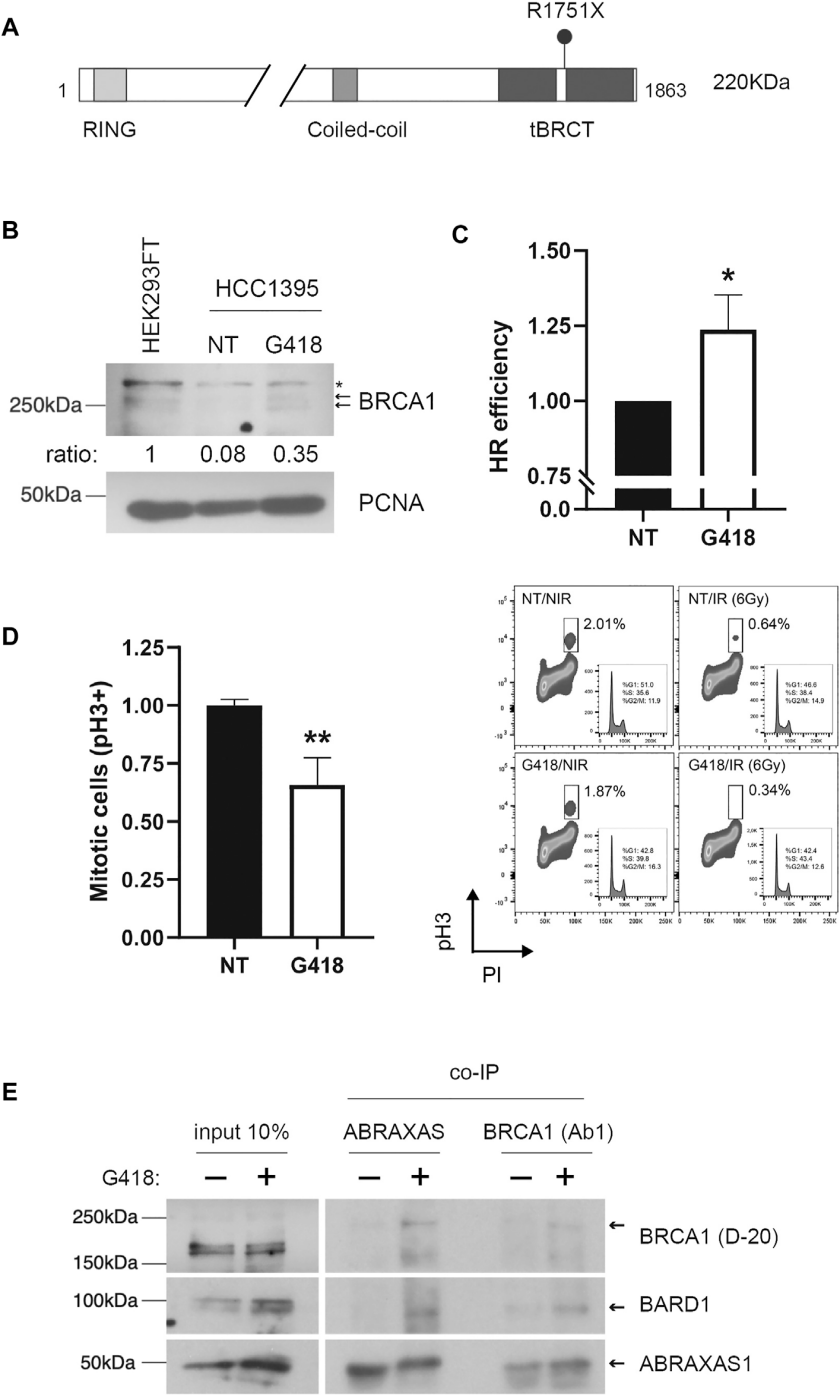
Aminoglycoside treatment restores BRCA1 FL protein expression and function in HCC1395 cells. **(A)** Schematic representation of BRCA1 protein depicting functional domains and R7151X variant position. Expected BRCA1 FL protein molecular weight: 220 KDa. **(B)** BRCA1 FL protein profile was assessed in nuclear cellular extracts of HEK293FT non-treated (NT) and HCC1935 cells either NT or treated with G418 (300 μg/ml) for 48 h. Arrows indicate BRCA1 phosphorylated forms and (*) indicates a non-specific band. PCNA was used as a loading control. Relative densitometric quantifications of BRCA1 bands are indicated (normalized to PCNA loading control). **(C)** Homologous recombination (HR) repair efficiency quantification was evaluated in HCC1935 cells NT or treated with G418. HR repair efficiency was analyzed by flow cytometry (mean of 3 independent experiments, error bars represent SD; *p < 0.05). **(D)** FACS analysis of PhosphoH3_ser10_ as a function of DNA content (PI) for HCC1395 cells NT or treated with G418 and then either non-irradiated (NIR) or irradiated (IR) with 6 Gy. Left panel: graphical representation of IR cells NT or G418-treated (mean of 3 independent experiments, error bars represent SD; **p < 0.005). Right panel: representative scatter plots. **(E)** Co-immunoprecipitation assays using anti-BRCA1 (Ab1) or anti-ABRAXAS1. HCC1395 cells were either NT or treated with G418, immunoblots were developed using BRCA1 (D-20), BARD1 and ABRAXAS1 antibodies; arrows indicate expected protein bands.

Geneticin (also known as G418) is one of the commonly used aminoglycosides antibiotics to evaluate stop-codon readthrough induction. To test whether we could induce PTC readthrough on the tumor suppressor gene *BRCA1*, we investigated the effect of G418 on HCC1395 human cells. This cell line carries the pathogenic *BRCA1* nonsense variant *p*. R1751X (c.5251C>T, generating UGA codon; NCBI Reference Sequence: NM_007294.4, ClinVar accession number: VCV000055480.10) in homozygosis (Gazdar et al., 1998) (Figure 1A). To assess readthrough induction, cells were treated with G418 for 48 h. As shown in Figure 1B, FL BRCA1 was observed only in HCC1395 treated cells, suggesting a pharmacologically-induced PTC readthrough. The immunoblotting analysis depicts phosphorylated forms of FL BRCA1 in the extracts of both HCC1395 treated cells and the BRCA1-proficient cell line HEK293FT, used here as a control for *BRCA1* WT expression (Cortez et al., 1999; Foo et al., 2021). As expected, the truncated protein was not detected, possibly due to the nonsense-mediated decay (NMD) system participation (Pawlicka et al., 2020).

To functionally interrogate *BRCA1* restoration, we examined the HR repair status using a specific I-SceI cleavage dependent GFP reporter assay in HCC1935 cells treated with G418. *BRCA1* is a key player in DDR, working as a platform for protein interaction that mediates HR and cell cycle control (Prakash et al., 2015). When treated with G418, HCC1395 cells exhibited an increase of 23.6% in HR repair efficiency when compared to untreated cells (Figure 1C), indicating a functional restoration of *BRCA1*, consistent with its FL protein recovery (Figure 1B). To further assess the functional impact of *BRCA1* readthrough induction we also tested whether restored *BRCA1* could promote cell cycle arrest in response to DNA damage. HCC1395 cells previously treated with G418 were exposed to IR, immunostained for phosphorylated histone 3^Ser10^ (phospho-H3), a marker of chromosome condensation during mitosis (Xu et al., 2002; Parsels et al., 2016), and co-stained with propidium iodide. Flow cytometry analysis was performed to quantify mitotic cells in non-IR and IR exposed conditions (Figure 1D). In response to DNA damage, HCC1395 G418/IR-treated cells displayed a well-characterized G2/M cell cycle arrest in BRCA1-proficient cells (Xu et al., 1999; Yarden et al., 2002), evidenced by a decrease in the mitotic cells population when compared to the impaired arrest exhibited by non-treated (NT)/IR cells. Normalized data indicate a ∼40% reduction in dividing cells, suggesting a partial restoration of G2/M cell cycle checkpoint associated with the readthrough event.

BRCA1 is found in distinct complexes in the DDR, such as in heterodimerization with BARD1, in BRCA1-A (CCDC98/ABRAXAS1, RAP80, BRCC36, BRCC45, MERIT40), BRCA1-B (TOPBP1 and BACH1/BRIP1), BRCA1-C (MRE11 and CtIP) and also in the BRCA1/PALB2/BRCA2 complex (Li and Greenberg, 2012; Savage and Harkin, 2015; Witus et al., 2022). These complexes play different roles in a context-dependent manner during DDR. BRCA1-A is associated with the recruitment of proteins to DNA damage sites in the initial steps of the repair pathway and G2/M checkpoint activation (Kim et al., 2007; Sobhian et al., 2007; Wang et al., 2007). The BRCA1/BARD1 heterodimer functions as a RING-type E3 ubiquitin ligase and is recruited to DSBs by the BRCA1-A complex (Witus et al., 2022). Thus, we inquired about the participation of restored BRCA1 in heterodimerization with BARD1 and BRCA1-A complex assembly. To answer this question, co-immunoprecipitation (co-IP) assays were conducted using nuclear extracts of HCC1935 cells treated or not with G418. In treated cells, ABRAXAS1 substantially co-immunoprecipitates BRCA1 as well as BARD1, contrasting with the untreated condition (Figure 1E). When performing the co-IP using anti-BRCA1 in the assay, ABRAXAS1 and BARD1 were recovered in the treated condition, similarly to what was observed in the ABRAXAS1 co-IP.

Altogether, these data show that the aminoglycoside G418 induces readthrough of a naturally occurring *BRCA1* nonsense mutation in a breast cancer cell line, restoring at some extent not only its FL form but also its function.

### *BRCA1* Restoration Levels Vary According to Premature Termination Codons Sequences

Stop codon readthrough inducing compounds facilitate near cognate amino-acyl tRNA incorporation leading to FL protein synthesis restoration and eventual functional recovery. The identity of the stop codon sequence has been reported to impact the readthrough efficiency. Previous reports have demonstrated that the likelihood of readthrough induction is greater for the UGA codon and lower for the UAA codon, with a consensus of readthrough likelihood being: UGA>UAG>UAA (Manuvakhova et al., 2000; Floquet et al., 2012; Dabrowski et al., 2015; Wangen and Green, 2020).

**FIGURE 2:**
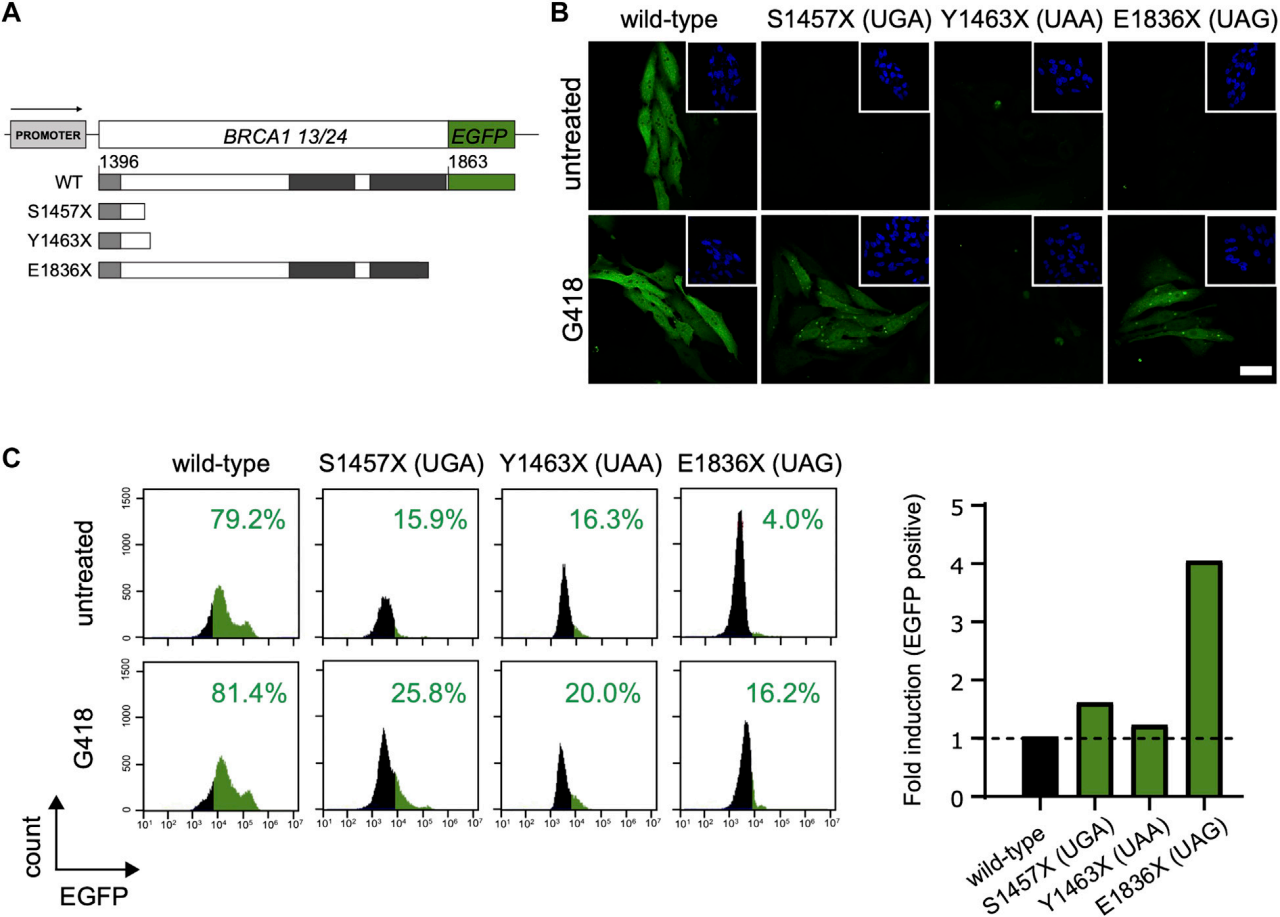
BRCA1 PTC readthrough evaluation in different stop codons. **(A)** Schematic representation of BRCA1-EGFP construct encoding amino acid residues 1,396 to 1,863 (corresponding to exons 13 to 24; BRCA1 13/24; upper panel). WT and PTC truncated proteins (lower panel) are represented; coiled-coil domain is indicated in light grey and the tBRCT indicated in dark grey. **(B)** Fluorescence confocal microscopy for HeLa BRCA1-EGFP cells encoding WT sequence or the different PTC variants. Cells were either untreated or treated with G418 (300 μg/ml) for 48 h and EGFP fluorescence was evaluated. Scale bar: 20 μM. **(C)** FACS analysis of HeLa BRCA1-EGFP cells encoding WT sequence or different PTC variants. Left panel: FACS histograms. Right panel: graphical representation of EGFP fold induction of G418 treated cells relative to untreated cells. Representative data from 3 independent experiments.

Thus, we questioned how different stop codons affect the induced readthrough of the tumor suppressor *BRCA1*. Readthrough was evaluated in three different naturally occurring BRCA1 PTCs: *p*. S1457X (c.4370C>G, coding UGA), *p*. Y1463X (c.4389C>A, coding UAA) and *p*. E1836X (c.5506G>T, coding UAG). HeLa BRCA1-EGFP cells, stably expressing the coding sequence for BRCA1 13/24 WT or the selected PTC variants fused to EGFP (Figure 2A) were used to examine protein restoration after aminoglycoside treatment. Restoration of BRCA1-EGFP is observed only in G418 treated cells (Figure 2B). Interestingly, confocal microscopy images indicate that restored EGFP levels vary among the different PTCs. HeLa S1457X (UGA) and HeLa E1836X (UAG) cells exhibited a more intense EGFP fluorescence, whereas HeLa Y1463X (UAA) presented discrete fluorescence. Flow cytometry analysis used to quantify EGFP positive cells in treated and non-treated conditions corroborated this observation (Figure 2C) and also validated the G418 readthrough induction in this model. HeLa E1836X (UAG) showed the highest fluorescence increase upon treatment (4-fold), HeLa S1457X (UGA) an increase of 0.6-fold, and HeLa Y1463X (UAA) displayed a modest 0.2-fold increase.

Indeed, the nonsense variant containing the termination codon UAA had the lowest readthrough induction efficiency, in accordance to the literature findings (Manuvakhova et al., 2000; Floquet et al., 2012; Dabrowski et al., 2015; Wangen and Green, 2020).

Our data suggest that G418 induces different *BRCA1* nonsense variants readthrough in different levels. Although, individually, other factors may impact each PTC readthrough efficiency.

### Readthrough Efficiency Depends on the Stop Codon Context

Besides the stop codon identity, the surrounding sequence contexts have been reported to influence pharmacological readthrough efficiency (Manuvakhova et al., 2000; Floquet et al., 2012; Dabrowski et al., 2015; Wangen and Green, 2020). This prompted us to evaluate further the stop codon sequence context to check whether other features influence *BRCA1* PTC readthrough. Evidence shows that, both in bacteria and eukaryotes, the identity of the nucleotide immediately after the stop codon (position +4, with the first nucleotide of the stop codon being +1) has great impact on readthrough efficiency (Manuvakhova et al., 2000; Floquet et al., 2012; Wangen & Green, 2020).

**FIGURE 3:**
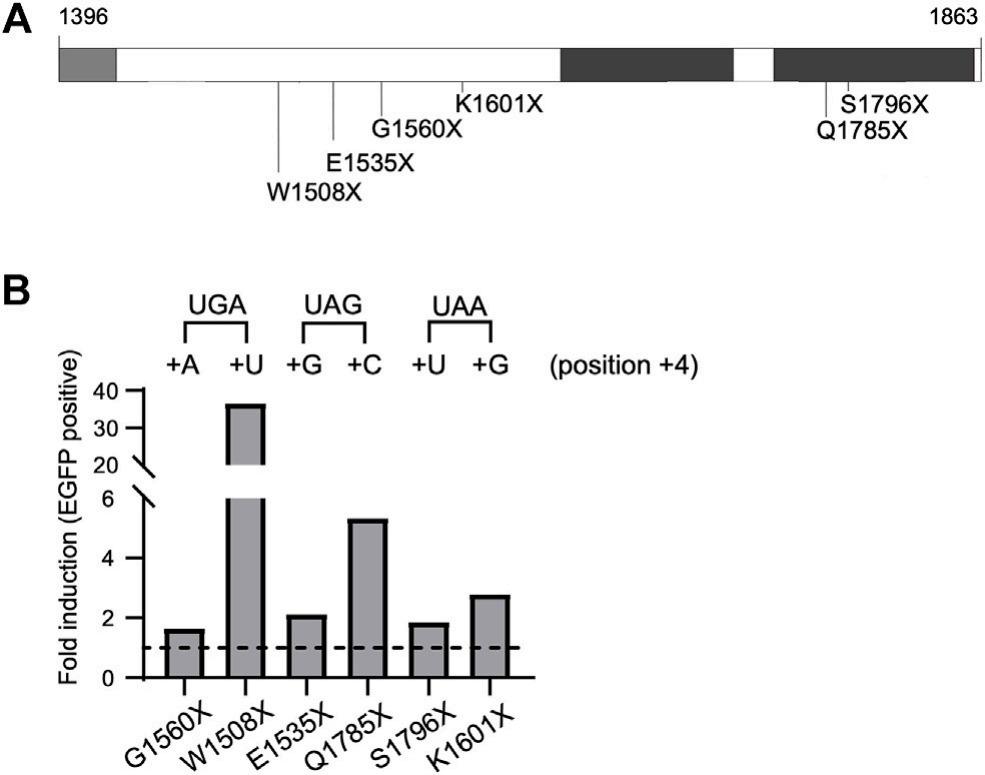
BRCA1 PTC readthrough induction depends on stop codon context. **(A)** Schematic representation of BRCA1-EGFP protein (amino acid residues 1,396–1,863), enclosing part of the coiled-coil (light grey) and the tBRCT (dark grey) domains, naturally occurring PTC variants analyzed are indicated. **(B)** FACS analysis of HeLa BRCA1-EGFP cells encoding different PTC variants. Cells were either non-treated (NT) or treated with G418 (300 μg/ml) for 48 h and EGFP fluorescence was evaluated. Graphical representation of EGFP fold induction in treated cells (relative to untreated cells). Termination sequences are indicated on top, nucleotides at position +4 are depicted. Representative data from 3 independent experiments.

To investigate the influence of the stop codon context on *BRCA1* PTC readthrough we interrogate a set of naturally occurring PTCs that displays different nucleotides at the position +4 using the HeLa BRCA1-EGFP model. BRCA1 PTC variants *p*. G1560X (c.4678G>T, coding UGA + A), *p*. W1508X (c.4524G>A, coding UGA + U), *p*. E1535X (c.4603G>T, coding UAG + G), *p*. Q1785X (c.5353C>T, coding UAG + C), *p*. S1796X (c.5387C>A, coding UAA + U), *p*. K1601X (c.4801A>T, coding UAA + G) are depicted in Figure 3A. After G418 treatment, HeLa cells stably expressing the selected PTC variants showed different extents of protein restoration (Figure 3B). UGA PTC variants expressing cells exhibited discrepant recovery rates among themselves: while HeLa G1560X (coding UGA + A) displayed a modest increase in EGFP positive cells upon treatment (1.6-fold), HeLa W1508X (UGA + U) exhibited a 36.6-fold increase. A moderate variation in fluorescence induction was observed for the UAG variants: HeLa E1535X (UAG + G) presented a 2.1-fold increase while HeLa Q1785X (UAG + C) displayed a 5.3-fold increase. On the other hand, the tested UAA PTC variants showed small variation in the rate of protein restoration when compared to the other PTCs: HeLa S1796X (UAA + U) exhibited a 1.9-fold increase and HeLa K1601X (UAA + G) a 2.8-fold increase.

Previous studies using report assays and genome-wide analysis have shown that, in general, having a pyrimidine (C/U) at position +4 favors PTC induced readthrough (Manuvakhova et al., 2000; Floquet et al., 2012; Wangen and Green, 2020). In agreement with these findings, the UGA + U and UAG + C BRCA1 PTC tested variants presented the highest readthrough rates.

Overall, we have shown that stop codon context, specifically the +4 nucleotide, impacts *BRCA1* PTC readthrough efficiency. These results support the idea that the PTC context, in particular the +4 nucleotide position, influences the efficiency of *BRCA1*-induced readthrough.

### Functional Characterization of Predicted Readthrough Proteins

PTC readthrough generates a FL protein that encodes an amino acid in substitution of the termination signal, most of the time leading to a missense mutation variant in this position (Wangen and Green, 2020). Roy and colleagues (2016) have described that in both yeast and human cells treated with G418 there are prevalent amino acid substitutions in the FL protein depending on the termination sequence. Treating human cells with G418 resulted in the insertion of Arg (∼64.5), Trp (∼17.9) and Cys (-17.7) at UGA codon; Gln (∼86.5), Tyr (∼10.8) and Lys (∼2.0) at UAG; and Gln (∼52) and Tyr (∼47.9) at UAA (Roy et al., 2016).

**FIGURE 4:**
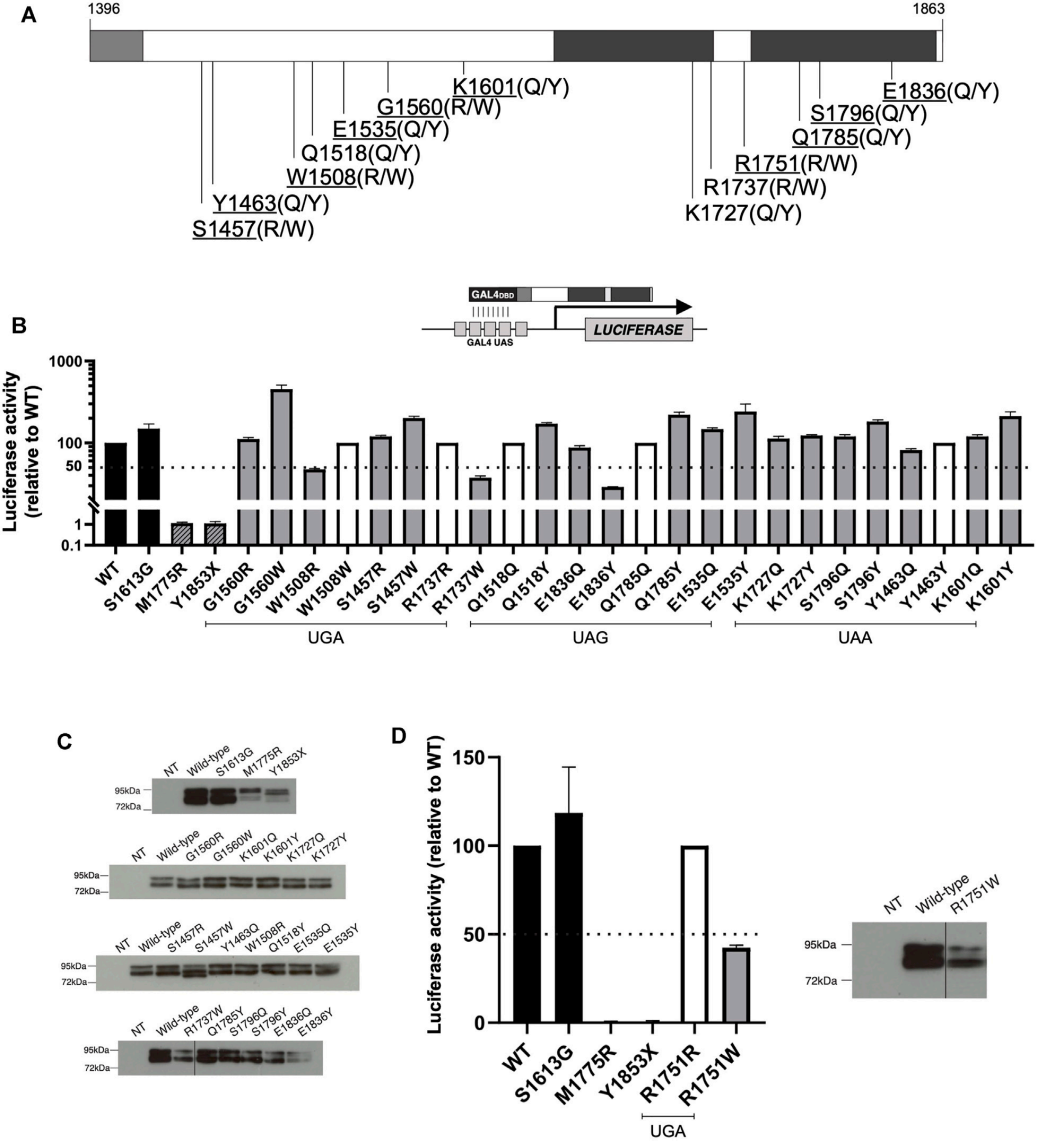
Functional characterization of expected prevalent BRCA1 missense variants generated after PTC readthrough. **(A)** Schematic representation of BRCA1 C-terminal region encoding amino acid residues 1,396 to 1,863, including part of the coiled-coil (light grey) and the tBRCT (dark grey) domains. WT amino acid residues related to the naturally occurring PTC variants positions are indicated and predicted prevalent amino acid substitutions are shown in parenthesis. The 10 nonsense variants positions that were assessed in this work are underlined. **(B)** TA assay for predicted readthrough BRCA1 missense variants encoded by GAL4-BRCA1 13/24 constructs was conducted in HEK293FT cells 24 h after transfection using a GAL4-responsive firefly luciferase reporter gene (inset scheme). Functional data are represented relative to WT (WT = 100%). WT and S1613G (black bars) were used as positive controls and M1775R and Y1853X as negative controls (dashed bars). Amino acid substitutions that restore WT sequence are represented by white bars (100% activity). 50% activity level is indicated by the dashed line. Mean and SD of 2 independent experiments. **(C)** GAL4-BRCA1 13/24 protein levels; immunoblot developed using α-GAL4 DBD antibody. **(D)** TA assay for predicted readthrough BRCA1 missense variants derived from R1751X PTC (as described for panel B). WT and R1751W GAL4-BRCA1 13/24 protein levels (as described for panel C). NT, non-transfected.

To better understand the effects of *BRCA1* PTC readthrough, we selected a set of 13 different nonsense mutations of natural occurrence at the C-terminal region of BRCA1 (10 of which were assessed in previous experiments in this work) and evaluated the functional impact of the resulting prevalent amino acid substitutions (Figure 4A), according to Roy and colleagues (2016) (Roy et al., 2016). Considering the first and second most prevalent substitutions identified for each stop codon, a total of 20 different missense mutation variants emerged from this analysis (Table 1). For 6 of the nonsense mutations (W1508X, R1737X, Q1518X, Q1785X, Y1463X, R1751X), one of the predicted prevalent amino acid substitutions would restore the wild-type protein sequence.

**TABLE 1:** Predicted readthrough variants - analyses compilation.

Nonsense variant	Stop codon	+4 nt	Expected codon readthrough substitution and frequency^*^	Readthrough variant #1	Align GVGD class	TA assay	Readthrough variant #2	Align GVGD class	TA assay
R1751X	UGA	G	Arg (∼64%), Trp (∼18%), and Cys (∼18%)	R1751R (WT)	–	NP	R1751W	C35	P
G1560X	UGA	A	Arg (∼64%), Trp (∼18%), and Cys (∼18%)	G1560R	C0	NP	G1560W	C0	NP
W1508X	UGA	U	Arg (∼64%), Trp (∼18%), and Cys (∼18%)	W1508R	C0	P	W1508W (WT)	–	NP
S1457X	UGA	C	Arg (∼64%), Trp (∼18%), and Cys (∼18%)	S1457R	C0	NP	S1457W	C0	NP
R1737X	UGA	G	Arg (∼64%), Trp (∼18%), and Cys (∼18%)	R1737R (WT)	–	NP	R1737W	C35	P
Q1518X	UAG	A	Gln (∼86%), Tyr (∼11%), and Lys (∼2%)	Q1518Q (WT)	–	NP	Q1518Y	C0	NP
E1836X	UAG	U	Gln (∼86%), Tyr (∼11%), and Lys (∼2%)	E1836Q	C0	NP	E1836Y	C15	P
Q1785X	UAG	C	Gln (∼86%), Tyr (∼11%), and Lys (∼2%)	Q1785Q (WT)	–	NP	Q1785Y	C0	NP
E1535X	UAG	G	Gln (∼86%), Tyr (∼11%), and Lys (∼2%)	E1535Q	C0	NP	E1535Y	C0	NP
K1727X	UAA	A	Gln (∼52%) and Tyr (∼48%)	K1727Q	C0	NP	K1727Y	C15	NP
S1796X	UAA	U	Gln (∼52%) and Tyr (∼48%)	S1796Q	C0	NP	S1796Y	C0	NP
Y1463X	UAA	C	Gln (∼52%) and Tyr (∼48%)	Y1463Q	C0	NP	Y1463Y (WT)	–	NP
K1601X	UAA	G	Gln (∼52%) and Tyr (∼48%)	K1601Q	C0	NP	K1601Y	C0	NP

* Roy et al., 2016.

NP, non-pathogenic; P, pathogenic.

We first evaluated putative readthrough missense variants *in silico* by the web-based tool Align-GVGD to predict their functional impact on protein biology. A score from C0 to C65 is given as a result, according to function interference probability (C0: less likely, C65: more likely). All of the most prevalent predicted substitutions (readthrough variant #1) that generate missense variants have a C0 Align-GVGD call (Table 1), suggesting that readthrough of the analyzed *BRCA1* PTC would mainly generate functional FL proteins.

To further characterize the functional impact of predicted *BRCA1* readthrough variants, we used a validated transcription activation (TA) based assay in human cells (Carvalho et al., 2007; Fernandes et al., 2019). Variants’ TA activity is compared to control readouts from the WT construct (100% activity), the benign polymorphism S1613G, the pathogenic missense variant M1775R and the pathogenic truncating mutation Y1853X. Predicted readthrough substitutions that restore wild-type sequence (100% activity) are represented by white bars and were included in the graphs for better visualization of all substitutions and their functional impact (Figure 4B). Immunoblot analysis suggests no significant changes in protein expression profile (Figure 4C). Only variants W1508R, R1737W and E1836Y displayed compromised functional activity (<50%), a characteristic of dysfunctional variants. Each of these variants correspond to one of the prevalent readthrough variants from nonsense mutations W1508X, R1737X and E1836X, respectively, and only variant W1508R would be the most frequent predicted substitution for its corresponding nonsense mutation (∼64% frequency, Table 1). Variants R1737W and E1836Y are the second most frequent substitution for each of their corresponding nonsense mutation (∼18 and ∼11% frequency, respectively). It is worth of note that the second most frequent readthrough product of W1508X would restore the wild-type sequence (∼18% frequency). Therefore, we conclude that for the analyzed BRCA1 PTC, readthrough induction would lead, primarily, to the generation of functional products, with the exception of W1508X PTC.

We have also analyzed the predicted amino acid substitutions for R1751X, the PTC observed in HCC1395 cell line (Figure 4D), as well as their functional impact. R1751X readthrough would most likely generate the wild-type sequence restoration (∼64% frequency). The missense variant R1751W would be the second most frequent readthrough product (∼18%), classified to have a moderate functional impact (C35) according to Align-GVGD (Table 1) and a pathogenic profile in TA assay (Figure 4D). Indeed, we have shown that R1751X readthrough levels were enough to restore other BRCA1 canonical functions (Figure 1C–E), in accordance to the prediction of readthrough inducing most likely the wild-type sequence restoration.

Taken together the results show that *BRCA1* PTC readthrough could lead to diverse substitution products depending on stop codon sequence, and that, for the PTC analyzed in this work, either one or both of the most frequent substitutions would lead to functionally active products. The functional characterization of predicted PTC readthrough products can help in the better comprehension of PTC readthrough induction overall impact.

## Discussion

The assessment of *BRCA1* status in hereditary breast and ovarian cancer patients is long established in the clinical routine to assist patients in prevention and treatment. The identification of *BRCA1* nonsense variants in patients confers a high risk of breast and ovarian cancer onset as these variants lead to protein truncation and loss of function (Hayes et al., 2000; Richards et al., 2015; Fernandes et al., 2019). For these individuals little can be done to a significant reduction in the cancer risk besides periodic monitoring routine and prophylactic surgeries. In theory, the restoration of BRCA1 function could be a pharmacological approach beneficial for such patients.

Here we introduce, for the first time, proof-of-concept data showing that the aminoglycoside G418 (geneticin) acts as a nonsense suppression treatment for *BRCA1* PTC *in vitro*. Not only G418 treatment is able to induce *BRCA1* PTC readthrough and, consequently, restoration of the FL protein, but also it restores BRCA1 function. For this purpose, we used as study models the breast cancer-derived cell line HCC1395, which carries the pathogenic nonsense variant R1751X in homozygosis, and a set of HeLa cells stably expressing BRCA1 C-terminal constructs carrying selected nonsense variants.

In the 1990s emerged the first indications that aminoglycosides could suppress nonsense mutations associated with diseases by readthrough induction (Allen and Raetz, 1992; Howard et al., 1996). Since then, many studies have evaluated aminoglycosides as a potential therapy targeting PTC mutations in genetic disorders to restore protein synthesis and function, *in vitro* and *in vivo* (Barton-Davis et al., 1999; Heier and DiDonato, 2009; Fuchshuber-Moraes et al., 2011; Lee and Dougherty, 2012; Kuschal et al., 2015). The encouraging results and the fact that some of these compounds were already used in the clinic, led to the conduction of clinical trials for genetic diseases such as DMD and CF, with observations of improved clinical status of treated patients (Wilschanski et al., 2003; Sermet-Gaudelus et al., 2007; Malik et al., 2010). Further efforts to find more effective and less toxic compounds identified PTC-124 as a PTC readthrough inducing agent, a non-aminoglycoside discovered in a screening among 800,000 compounds (Welch et al., 2007). The positive pre-clinical results prompted the drug to be tested in many clinical trials resulting in mild clinical improvements in some of them (Martins-Dias and Romão, 2021).

Nonsense suppression therapy has also been explored in cancer-causing genetic disorders, reviewed by (Lee and Dougherty, 2012; Martins-Dias and Romão, 2021). Induction of PTC readthrough in the *TP53* gene was shown in different approaches, and despite mild protein expression restoration levels, its biological activities and tumor suppressor function were restored (Floquet et al., 2011). Restoration of FL protein synthesis and function was also observed in mice, human cells, and clinical models for *APC* and *XPC* genes (Zilberberg et al., 2010; Kuschal et al., 2013, 2015).

Here we show that G418 induces readthrough of *BRCA1* PTC mutation R1751X (UGA + G code) carried by HCC1395 human cell line. This was also accompanied by the restoration of HR capacity, G2/M cell cycle arrest upon DNA damage, formation of the BRCA1/BARD1 and BRCA1-A complex, critical to BRCA1 functions for tumor suppression (Figure 1) (Tarsounas and Sung, 2020; Witus et al., 2022). There is a consensus in the literature that UGA has the greatest likelihood of being readthrough, followed by UAG and lastly by UAA (Manuvakhova et al., 2000; Floquet et al., 2012; Dabrowski et al., 2015; Wangen and Green, 2020). Evidence also shows that UGA stop codon, when readthrough, is most likely misread as arginine (Roy et al., 2016), which in the case of R1751X would restore BRCA1 wild-type sequence. Collectively, these data might explain the encouraging results presented here.

We further investigated whether the identity of other BRCA1 naturally occurring PTCs would affect G418 readthrough induction. We used a HeLa BRCA1-EGFP model stably expressing BRCA1 C-terminal EGFP constructs and EGFP fluorescence as our readthrough reporter (Figure 2). We found that PTC readthrough induction varied among different stop codon contexts. W1508X (UGA + U) variant showed the highest EGFP fluorescence levels in response to G418 treatment (Figure 3). This variant, like R1751X, is also coded by UGA, favoring readthrough. The nucleotide immediately after the stop codon (position +4), also affects readthrough efficiency (Manuvakhova et al., 2000; Floquet et al., 2012; Wangen and Green, 2020). In combination with the identity of the stop codon triplet, a pyrimidine (C/U) at position +4 favors PTC-induced readthrough. Thus, W1508X variant would combine a favorable four-nucleotide code for readthrough induction. Variant Y1463X (UAA + C) displayed the lowest EGFP rate response (Figure 2), in accordance with literature description that UAA has the lowest readthrough likelihood. All UAG codon variants analyzed (E1535X, Q1785X, E1836X) demonstrated an intermediary impact in readthrough, in consonance with literature description, as mentioned above. (Figures 2, 3).

Nonetheless, not all of the analyzed variants showed readthrough activity as demonstrated in previous literature findings. For example, variants G1560X (UGA + A) and S1457X (UGA + C) demonstrated low readthrough activities despite having the most favorable readthrough codon (UGA). This leads us to suggest that the identity of the codon triplet and +4 position are not the only determining features of *BRCA1* PTC readthrough efficiency. Indeed, it has been shown that the vicinity upstream and downstream to the stop codon triplet can influence termination fidelity and readthrough induction, such as -2, -1, +5 and +6 positions, and also mRNA levels and availability of termination machinery components (reviewed in Lee and Dougherty, 2012; Dabrowski et al., 2015). However, further studies are necessary in order to better understand the impact of *BRCA1* PTC sequence and vicinity in readthrough induction.

Our results show that *BRCA1* PTC variants can be suppressed by G418 treatment, but whether protein function is restored depends on the prevalent amino acids incorporated due to PTC readthrough induction (Roy et al., 2016; Xue et al., 2017). We combined our validated BRCA1 transcriptional activation (TA) assay and an *in silico* method (Align-GVGD) to evaluate the functional impact of predicted amino acid substitutions (Figure 4). We observed that amino acid substitutions would lead primarily to functional products, except for W1508X (substituted primarily by W1508R) - curiously, this PTC variant presented the highest readthrough induction level. Overall, functional impact predicted by both *in silico* and *in vitro* methods showed a good correlation, where C0 *in silico* classification coincided mainly with non-pathogenic *in vitro* results.

In conclusion, we show that G418 induces *BRCA1* PTC readthrough with variable efficiencies among different stop codon contexts. The data indicate that *BRCA1* PTC readthrough products are predominantly functional and functional evaluation of predicted induced substitutions can be informative to assess readthrough overall impact. Further studies, including evaluation of other nonsense suppressor agents and *in vivo* approaches, are critical to improve our understanding of *BRCA1* PTC readthrough and consider its potential for clinical applications.

## Data Availability

The original contributions presented in the study are included in the article/Supplementary Material, further inquiries can be directed to the corresponding author.
